# Supplementary material for: Transcriptome Profiling of Spike Development Reveals Key Genes and Pathways Associated with Early Heading in Wheat–Psathyrstachys huashanica 7Ns Chromosome Addition Line
Source: Plants (Basel). 2025 Jul 7;14(13):2077. doi: 10.3390/plants14132077 (PMC12252408; doi:10.3390/plants14132077)
Supplement: Supplementary file 1 [file plants-14-02077-s001.zip › Supplementary Figures.pdf]

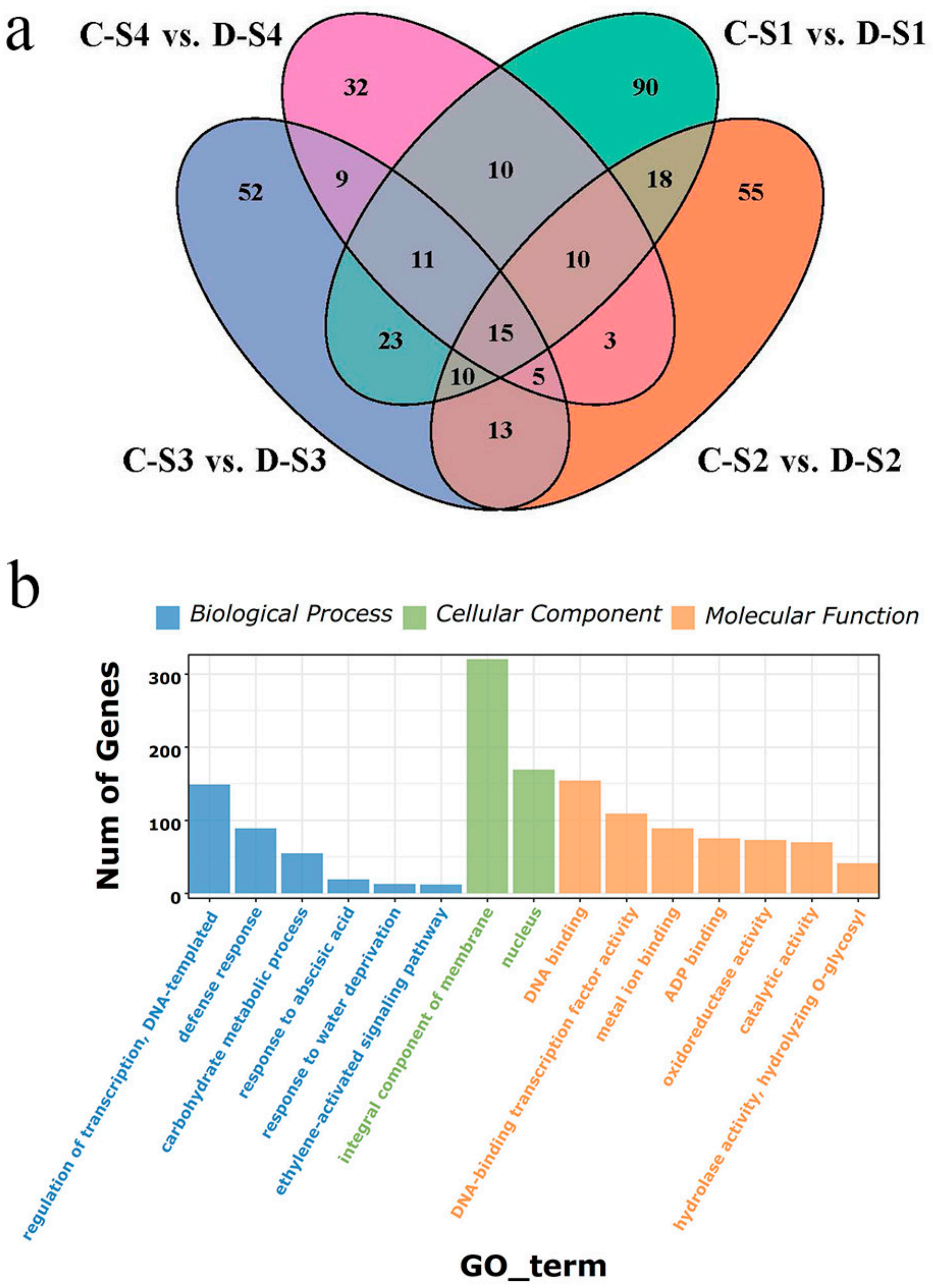

Figure S1 Common GO terms in four different comparison groups. (**a**) Venn diagram showing the number of common GO terms in all the comparison groups. (**b**) Histogram of common GO terms in all the comparison groups. The capital letter "C" represents 18-1-5, and "D" indicates CS and CS*ph2b*. S1, S2, S3, and S4 represent the double-ridge stage, the glume primordia differentiation stage, the floret primordia differentiation stage, and the stamen and pistil differentiation stage, respectively.

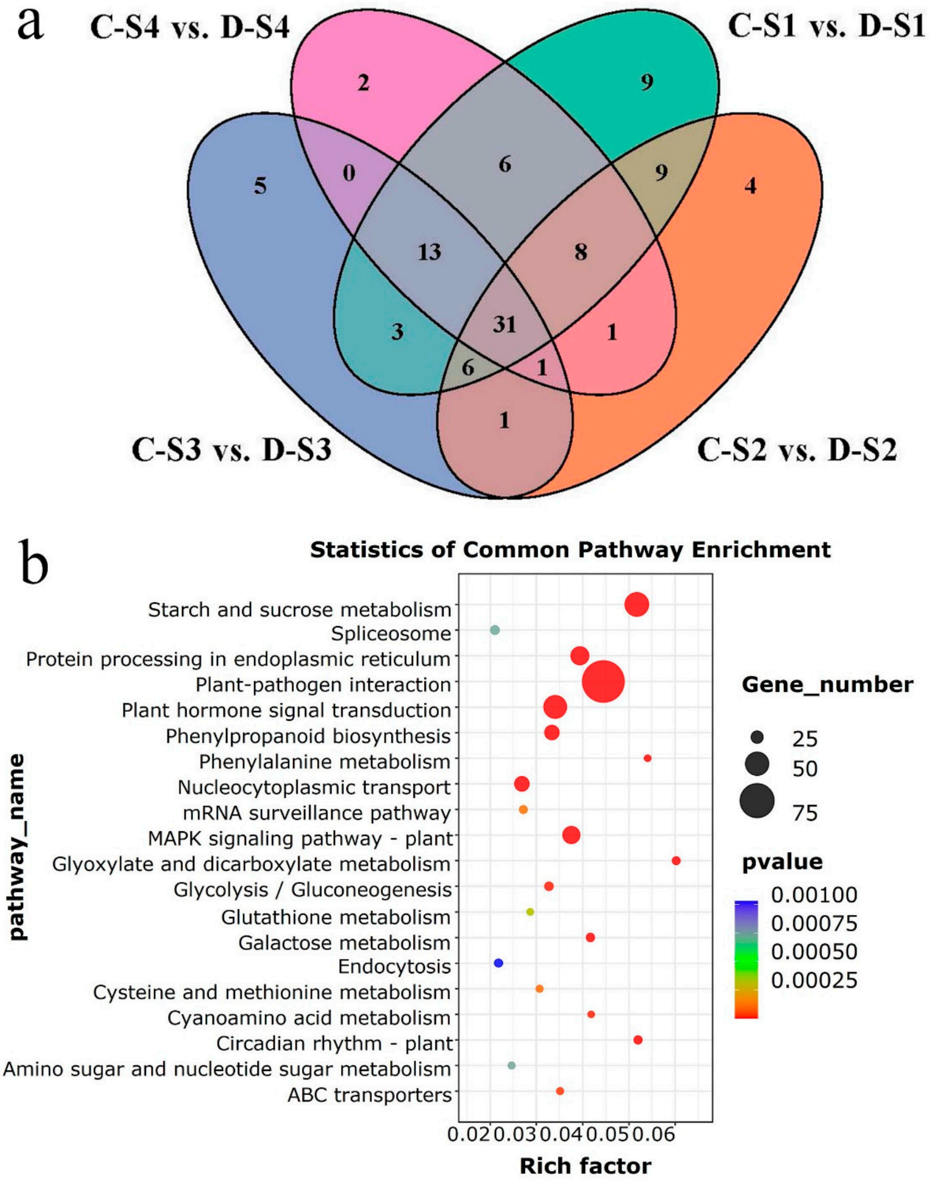

Figure S2 Common KEGG enrichment pathways in four different comparison groups. (**a**) Venn diagram showing the number of common KEGG enrichment pathways in all the comparison groups. (**b**) A scattered plot of KEGG enrichment pathways in all the comparison groups. The capital letter "C" represents 18-1-5, and "D" indicates CS and CS*ph2b*. S1, S2, S3, and S4 represent the double-ridge stage, the glume primordia differentiation stage, the floret primordia differentiation stage, and the stamen and pistil differentiation stage, respectively.
